# The actin crosslinking protein palladin modulates force generation and mechanosensitivity of tumor associated fibroblasts

**DOI:** 10.1038/srep28805

**Published:** 2016-06-29

**Authors:** Mikheil Azatov, Silvia M. Goicoechea, Carol A. Otey, Arpita Upadhyaya

**Affiliations:** 1 Department of Physics, University of Maryland, College Park MD 20742, USA; 2 Department of Biological Sciences, University of Toledo, Toledo, Ohio, 43606, USA; 3 Department of Cell Biology and Physiology and the Lineberger Comprehensive Cancer Center, University of North Carolina at Chapel Hill, Chapel Hill, NC 27599, USA; 4 Institute for Physical Science and Technology, University of Maryland, College Park, MD 20742, USA

## Abstract

Cells organize actin filaments into higher-order structures by regulating the composition, distribution and concentration of actin crosslinkers. Palladin is an actin crosslinker found in the lamellar actin network and stress fibers, which are critical for mechanosensing of the environment. Palladin also serves as a molecular scaffold for α-actinin, another key actin crosslinker. By virtue of its close interactions with actomyosin structures in the cell, palladin may play an important role in cell mechanics. However, the role of palladin in cellular force generation and mechanosensing has not been studied. Here, we investigate the role of palladin in regulating the plasticity of the actin cytoskeleton and cellular force generation in response to alterations in substrate stiffness. Traction force microscopy revealed that tumor-associated fibroblasts generate larger forces on substrates of increased stiffness. Contrary to expectations, knocking down palladin increased the forces generated by cells and inhibited their ability to sense substrate stiffness for very stiff gels. This was accompanied by significant differences in actin organization, adhesion dynamics and altered myosin organization in palladin knock-down cells. Our results suggest that actin crosslinkers such as palladin and myosin motors coordinate for optimal cell function and to prevent aberrant behavior as in cancer metastasis.

Many aspects of cell behavior are dependent on the physical properties of a cell’s environment^1^,^2^. Cell migration is susceptible to the mechanical properties of the environment such as substrate elasticity^3^. Stem cell differentiation into different cell types is modulated by the elasticity of the microenvironment^4^. It is becoming increasingly clear that the mechanical interactions of cancer cells with their environment are essential components in tumor progression and metastasis^5^,^6^.

The molecular mechanisms that enable cells to sense and respond to the mechanical properties of their environment are being intensely studied^7^. The cytoskeleton and cell adhesions are key components that enable cells to sense their mechanical environment. Extensive work has shown that focal adhesions act as mechanosensors^8^,^9^,^10^,^11^,^12^. In accord with this, the size, morphology and dynamics of focal adhesions depend on matrix stiffness^13^,^14^,^15^. The coupling of focal adhesions to actin filaments enables myosin motors to exert forces and transmit contractile tension to the substrate allowing the cell to sample the substrate stiffness. Actin crosslinking proteins which link actin filaments with developing adhesions and the extracellular matrix, and which organize actin filaments into large-scale coherent structures are important for force generation^8^. However, their contribution to mechanotransduction is only now being understood^16^,^17^,^18^.

Most mammalian cells express a diverse array of actin crosslinking proteins. The contribution of crosslinkers in organizing actin networks has been examined for crosslinkers such as α-actinin and zyxin^19^,^20^,^21^,^22^,^23^. α-actinin is involved in force transmission to the ECM via integrin binding^21^, while zyxin is important in maintenance of stress fiber integrity under applied loads^22^. The actin-binding protein, palladin, occupies a unique molecular niche, functioning as a molecular scaffold that directs the assembly and organization of actin networks^24^. Palladin directly binds actin filaments through its multiple Ig (Immunoglobulin-like) domains^25^, binds to the actin crosslinker, α-actinin, and colocalizes with α-actinin along stress fibers^26^,^27^,^28^. *In vitro* assays show that palladin crosslinks actin into viscoelastic networks and synergistically combines with α-actinin^29^. Palladin is up-regulated in pancreatic tumor-associated fibroblasts (TAFs) which have been shown to promote the progression of pancreatic tumors, metastasis, and resistance to therapy^30^,^31^,^32^. Evidence suggests that the misregulation of actin reorganization resulting from altered palladin levels may contribute to aberrant cellular behavior. Given its localization in the cell, it is a likely candidate for force transmission. However, the role of palladin in focal adhesion maturation and actin organization for force transmission and cell response to ECM properties, such as stiffness, is unclear.

Here, we use pancreatic TAFs to examine the role of palladin in actin organization, force generation and mechanosensing. As a model to study mechanosensing, TAFs are of particular interest because of their complex role in the assembly and dynamic remodeling of the tumor stroma^33^,^34^. We found that palladin plays a role in adhesion maturation, stress fiber formation and actin flows, and has a significant effect on cellular forces. Our experiments also suggest that palladin may have an effect on myosin activity and organization in cells. Taken together, our results demonstrate an important role for palladin in regulating cellular forces and mechanosensing.

## Methods

### Cell culture, transfection and immunostaining

The palladin knockdown (KD) cell line (Palld4) in which palladin was silenced using shRNA sequence and the scrambled siRNA control (PGIPZ), were created as described previously^35^. Quantitative Western blots showed that palladin levels were reduced by 90% in the Palld4 line^35^. Wildtype, EGFP-palladin, KD and shRNA control cells were cultured in DMEM with 10% FBS, 1% PS and sodium pyruvate at 37 °C. For spreading experiments, cells were plated at 15% confluence on fibronectin (from bovine plasma, Sigma-Aldrich) coated glass coverslips. Coverslips were incubated with with 500 μl of 10 μg/ml fibronectin solution for 2 hours at room temperature. Imaging media L-15 (Life technologies, Grand Island, NY) was used for microscopy. For actin visualization, cells were fixed using paraformaldehyde and stained with rhodamine-phalloidin. Transient transfections were done with mApple-paxillin, mCherry-actin, mCherry-MHC-IIA (myosin heavy chain) using Fugene HD tranfection reagent (Promega, Madison WI) and manufacturer protocol. For immunostaining, cells were fixed with 4% paraformaldehyde (PFA) solution for 7 minutes, washed with phosphate buffered saline (PBS) and permeabilized with 0.2% solution of Triton-X for 2 minutes. They were then washed with PBS and blocked (2% BSA in PBS) for 1 h. Cells were incubated with primary antibody (Myosin IIa antibody), in blocking solution for 1 h, washed in PBS and incubated in secondary antibody solution (Alexa Fluor 546 goat anti-rabbit, Invitrogen A11010) for 1 h in the dark.

EGFP-palladin fragment was ligated into Z4-MSCV-mEos2-actin (a gift from Morgan Huse, Rockefeller University, New York, NY). Retroviruses were generated according to standard protocol^36^, with Phoenix Amphotropic cells and transduced into TAFs. The cells were then selected in 100 μg/ml zeocin for 2 weeks and sorted with fluorescence-activated cell sorting to obtain fluorescent cells. Palladin showed a 2X overexpression in EGFP-palladin cells compared to WT cells based on quantitative Western blot (data not shown).

### Traction forces and preparation of PAA gels

For traction force experiments fibronectin-coated polyacrylamide (PAA) gels with fluorescent beads on the top layer were prepared as before^37^,^38^. The ratio of 40% acrylamide to 2% BIS (Bio-Rad, Hercules, CA) was varied (2:0.1, 3:0.1, 4:0.1, 5:0.1) to obtain gels of different stiffness ranging from 1 kPa–60 kPA. Glass coverslips were coated with 3-aminopropyl-trimethocysilane and glutaraldehyde (Sigma-Aldrich, St. Louis, MO) to allow the polymerizing gels to conjugate to the surface. A thin (5 μm) layer of gel with 200 nm diameter fluorescent beads was attached to the top surface. 500 μl of sulfo-SANPAH (ProteoChem, Loves Park, IL) solution was added to the top of the gel and incubated in the dark for 30 minutes. The sulfo-SANPAH was washed away with PBS and 500 μl of fibronectin solution (10μg/ml) was pipeted onto the gel and placed 2 inches below an 8 W UV lamp for 8 minutes.

After obtaining images of multiple cells and corresponding beads on a gel, cells were detached by trypsinization to obtain a reference (or zero displacement) image for traction force analysis. After each imaging experiment, the gel height was determined using the microscope’s z-focus mechanism and corrected for axial scaling. Typical gel heights were 68 ± 12 *μm*. The Young’s modulus of each gel was measured using the stainless steel ball indentation method^39^ rather than using a relation between gel stiffness and BIS concentration since the measured modulus varied from gel to gel even with the same formulation.

After drift correction, the displacement of fluorescent beads between two images (corresponding to the deformed and undeformed gel) was calculated using particle image velocimetry (PIV) (using the open-source MATLAB package MPIV, http://folk.uio.no/jks/matpiv/index2.html). The window size for the adaptive PIV algorithm ranged from 8 × 8 to 64 × 64 pixels with an overlap of 50%, yielding a resolution of ~2–3 *μ*m. Displacement vector maps were input into an unconstrained Fourier Transform Traction Cytometry (FTTC) algorithm implemented in MATLAB^40^ and extended to include finite thickness correction^41^. FTTC analysis was used to obtain the traction stress magnitude and vector maps. The total force exerted by the cell was calculated using 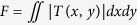, where *T(x, y)* is the stress at location *x,y*.

### Live cell microscopy

Fluorescence and Interference Reflection Microscopy images were collected at 37 °C using an inverted microscope (TE2000 PFS, Nikon, Melville, NY) with a cooled CCD camera (Coolsnap HQ2, Photometrics, Tucson, AZ). TIRF imaging was done using a 60x magnification, 1.49 NA objective lens, a 491 nm laser (100 mW, Andor, South Windsor, CT) for EGFP excitation and a 561 nm laser (75 mW, Andor) for mApple and mCherry excitation.

For blebbistatin recovery experiments, cells were allowed to spread on fibronectin-coated gels for 3 hours and imaged. 15 μM blebbistatin was added to the imaging chamber and incubated for 30 min. Blebbistatin was then washed out and replaced with imaging medium while cell recovery was monitored. Time-lapse imaging of cells and beads throughout the washout and recovery process enabled traction forces to be computed. Finally, cells were detached by trypsinization to obtain reference bead images.

### Image Analysis

To quantify focal adhesions, polygons were drawn around maturing focal adhesions. Focal adhesion length was defined as the diagonal of the rectangle around a polygon. The focal adhesion maturation time was defined as the time taken for the mean fluorescence intensity in the defining polygons to reach its maximum value from the onset of the rise. Radial Stress Fibers were marked by their location as in Oakes *et al*.^20^. RSF were identified by eye using rhodamine-phalloidin actin staining 4h after spreading and defined as stress fibers that are roughly perpendicular to the cell boundary.

### Statistical analysis

Since many of the measured parameters were not Gaussian-distributed, we used the non-parametric Wilcoxon’s rank-sum test to determine the significance with respect to WT. The resulting *p*-values are indicated on the figures and in the figure legends. Error bars denote SEM (standard error of the mean).

## Results

### Palladin is important for focal adhesion maturation and radial stress fibers

**Figure 1:**
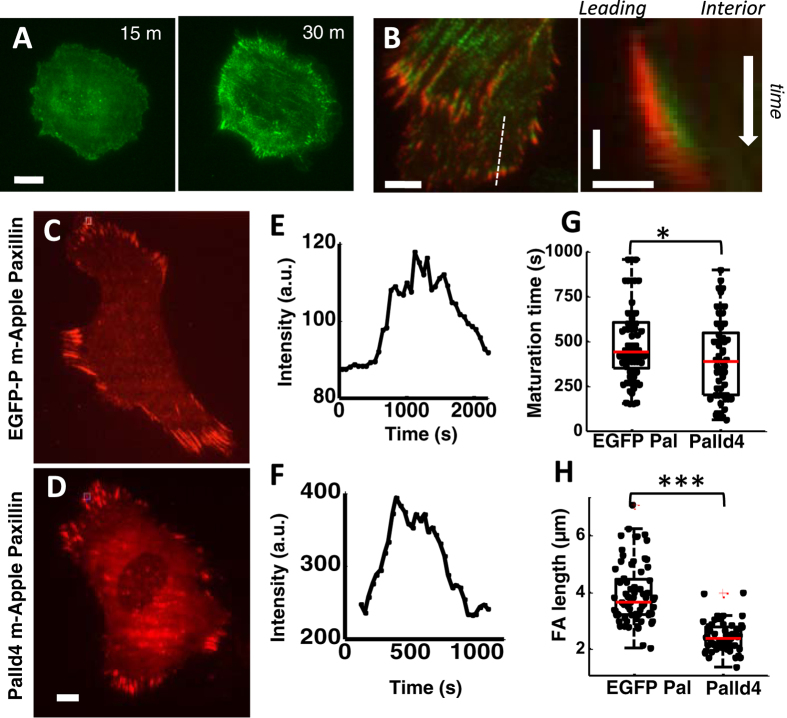
Palladin associates with focal adhesions and modulates focal adhesion maturation. **(A)** TIRF images of EGFP-palladin cell spreading on glass showing palladin organization as it changes from a diffuse localization into mature adhesions and stress fibers. Scale bar: 15 μm. **(B)** Left panel: Dual color image of a cell expressing EGFP-palladin (green) and mCherry-paxillin (red) showing focal adhesions and stress fibers. Scale bar: 5 μm. Right panel: Kymograph along the direction of growth of a focal adhesion (as indicated by white line in the left), showing accumulation of paxillin and palladin in a focal adhesion. Scale bars: 3 μm horizontal, 2 min vertical. TIRF image of **(C)** EGFP-palladin cell and **(D)** Palladin knockdown (Palld4) cell transfected with mApple-paxillin (red) showing multiple focal adhesions along the cell periphery. Scale bar: 15 μm. Plot of the mean fluorescence intensity of a focal adhesion as a function of time during adhesion maturation in (**E**) an EGFP-palladin cell and **(F)** a palladin knockdown cell. **(G)** Beeswarm graphs showing comparison of the maturation times of focal adhesions in EGFP-palladin and Palld4 cells (p < 0.0001, Wilcoxon ranksum test). **(H)** Comparison of focal adhesion length in EGFP-palladin and Palld4 cells (p < 0.01, Wilcoxon rank sum test). The box shows the interquartile range of the data, spanning from first quartile to the third quartile of the data, red line indicates the median and whiskers denote 1.5x interquartile range.

We first examined the distribution of palladin in TAFs expressing green fluorescent protein tagged palladin (EGFP-palladin) and visualized the cell morphology as it spread on fibronectin-coated glass coverslips using total internal reflection fluorescence (TIRF) (Fig. 1A). During early spreading, palladin appeared diffusely in the contact zone or formed highly mobile puncta that assembled into nascent adhesions at the cell periphery. After 30 min of spreading, when the spread area was maximal, palladin puncta organized to form mature focal adhesions and palladin was recruited into assembling stress fibers. Upon completion of spreading, palladin underwent a constant retrograde flow from the cell edge towards the interior along stress fiber templates. Rhodamine-phalloidin staining to visualize filamentous actin (f-actin) simultaneously with EGFP-palladin showed that palladin colocalized with actin and formed punctate spots along stress fibers, as observed before (Supplementary Fig. S1)^24^,^28^,^42^,^43^.

Mechanosensing is believed to arise from the tension-mediated maturation of focal adhesions^13^,^14^,^15^,^44^. To examine the effect of palladin expression on focal adhesion formation and dynamics, we constructed a cell line knocked down in palladin (Palld4) in which palladin expression levels were reduced by ~90% (see Methods)^35^. We used paxillin, a key component of adhesions in cells, as a focal adhesion marker. We transfected EGFP-palladin and Palld4 cells with mApple-paxillin and used dual-wavelength TIRF to visualize focal adhesion formation. Paxillin primarily localized to the tips of adhesions while palladin appeared in adhesions and stress fibers (Fig. 1B left). During early spreading, palladin localized to a thin region at the cell periphery, while paxillin appeared next to palladin towards the cell interior. Focal adhesions grew towards the interior as shown in the kymograph (Fig. 1B right). To examine whether palladin expression affects focal adhesion maturation, we measured focal adhesion length and timescale of formation in EGFP-palladin and Palld4 cells (Fig. 1C,D). The fluorescence intensity of maturing adhesions rapidly increased as adhesions formed and grew (Fig. 1E,F). Palladin knockdown resulted in shorter focal adhesions with a smaller maturation time (Fig. 1G,H). This indicates a role for palladin in focal adhesion templating and growth.

**Figure 2:**
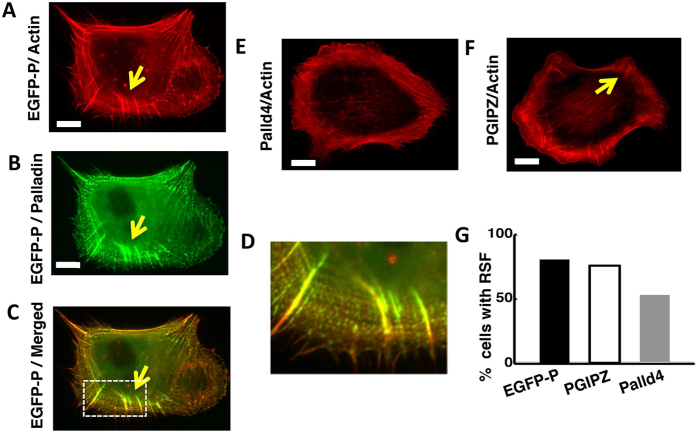
Palladin knockdown impairs radial fiber formation. **(A)** Snapshot of EGFP-palladin cell labeled with Rhodamine-phalloidin for actin showing strong radial fibers (RSF). A typical RSF is indicated by the arrow. Scale bar: 15 μm. **(B)** Snapshot of EGFP-palladin cell showing localization of palladin in RSF. Scale bar: 15 μm. (**C)** Merged image showing co-localization of actin and palladin in RSF. (**D**) Zoomed in image of the region the dotted boxed from (**C**) showing RSF in greater detail. **(E)** Snapshot of palladin KD cells (Palld4) labeled with Rhodamine-phalloidin showing a lack of radial stress fibers in the cell. Scale bar: 15 μm. **(F)** Snapshot of a control shRNA cell (PGIPZ) showing the presence of radial fibers as indicated by the arrow for an example. Scale bar: 15 μm for all panels. **(G)** Bar graph showing comparison of the percentage of cells (EGFP-palladin, control sh-RNA (PGIPZ), and palladin KD (Palld4)) which displayed radial fibers quantified at 4 hours after spreading initiation (*N* = 30 for each condition).

We next examined whether palladin knockdown affected actin organization, specifically stress fiber formation. A wide variety of adhesive and contractile cells possess actin stress fibers, which have been postulated to play an important role in the transmission of cellular forces^45^,^46^. Since focal adhesions are known to serve as templates for stress fiber assembly^19^,^20^, we hypothesized that palladin is important for stress fiber formation in TAFs. We allowed EGFP-palladin, Palld4 and cells with control sh-RNA (PGIPZ) to spread on FN coated coverslips, fixed the cells after 4 hours of spreading, and stained with Rhodamine-phalloidin to visualize f-actin. EGFP-palladin cells displayed radial stress fibers (RSF) enriched in both f-actin and EGFP-palladin (Fig. 2A–D), visible as bright structures roughly oriented radially with respect to the cell edge. Palld4 cells showed significantly fewer RSF (Fig. 2E). Further example images of both types of cells are shown in Supplementary Fig. S2.

Cells with control sh-RNA (PGIPZ) also displayed robust RSF formation (Fig. 2F). We scored cells as having RSF if they had more than five RSF. We found that a greater fraction of EGFP-Palladin cells have RSF as compared to palladin KD cells (Fig. 2G), consistent with previous results with α-actinin^20^,^21^. The average number of RSF per cell was measured to be 13.9 ± 1.7 and 7.6 ± 2.2 for EGFP-palladin and Palld4 respectively (*N* = 30 cells of each type). We observed qualitatively that RSF have enhanced levels of EGFP-palladin fluorescence, consistent with previous observations of increased F-actin in RSF^20^,^21^. These observations show that EGFP-palladin cells have a greater ability to form RSF confirming palladin’s role in RSF formation.

### Tumor-associated fibroblasts are mechanosensitive

Actin crosslinkers have been implicated in the regulation of cellular forces^16^,^17^,^18^. Our findings that palladin is a regulator of focal adhesion maturation and RSF formation, and the postulated role of RSF in force transmission from cells to ECM via focal adhesions, led us to surmise that palladin may be critical for cellular force generation and mechanosensing. We therefore investigated palladin’s role in cellular forces.

**Figure 3:**
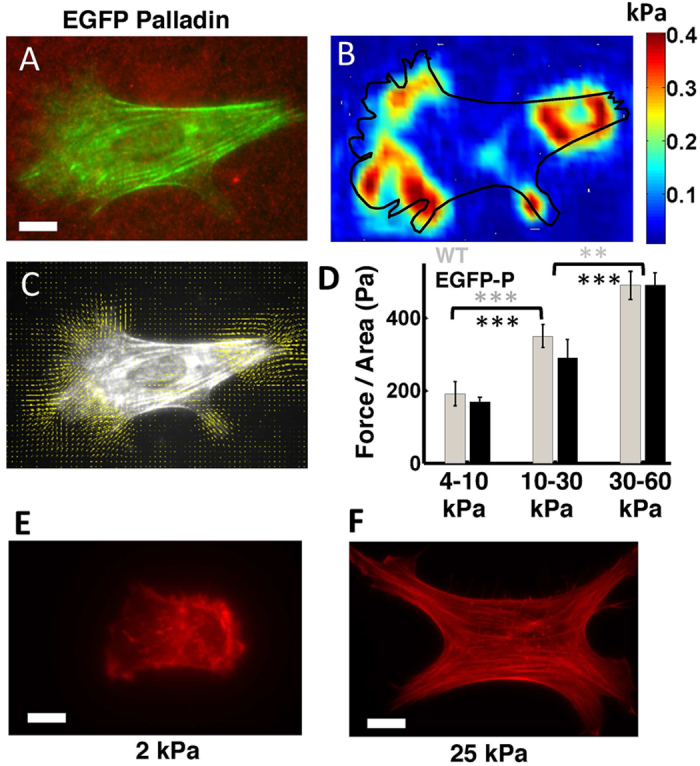
Tumor-associated fibroblasts are mechanosensitive. **(A)** Snapshot of an EGFP-palladin cell (green) on an elastic gel (in the 10–30 kPa stiffness range) embedded with fluorescent beads (red). Scale bar: 10 μm. **(B)** Snapshot of the traction stress map for the stresses generated by the same cell. Colors correspond to the stress values as indicated by the color scale. The contour of the cell is superimposed in black. **(C)** A map of the local traction force vectors superimposed on the cell contour. **(D)** Traction force per unit area for WT and EGFP-palladin cells on gels of different stiffness ranges. (WT cells: grey: p < 0.001 between soft and intermediate; p < 0.01 between intermediate and stiff; Wilcoxon ranksum test) (EGFP-palladin cells: black: p < 0.0001 between soft and intermediate; p < 0.0001 between intermediate and stiff; Wilcoxon ranksum test). Number of cells: *N* > 20 from ~5 independent experiments for each condition (corresponding to a total of ~120 cells). Rhodamine-phalloidin staining of WT cell to visualize f-actin on **(E)** a soft (2 kPa) gel and **(F)** a stiffer (25 kPa) gel. Scale bars: 10 μm.

We examined the response of TAFs to varying matrix stiffness using traction force microscopy. Cells were allowed to spread on fibronectin-coated polyacrylamide gels for 3–4 h and then imaged with wide-field fluorescence microscopy. Fluorescence images of EGFP-palladin cells in the green channel, or bright-field images of wild-type TAF cells were obtained simultaneously with bead images in the red channel (Fig. 3A). Traction forces were obtained from the displacement of beads on elastic gels deformed by cellular forces (see Methods for details). Figure 3B shows a representative map of traction stresses generated by the cell in 3A. Typical peak tractions were on the order of a few hundred Pa, consistent with observations in other cells^47^. A representative vector map of traction forces superimposed on the cell’s fluorescence image shows centripetal or inward directed tractions (Fig. 3C). The majority of stress was exerted in the cell periphery with strong cell-substrate attachments.

To examine the effect of substrate stiffness on cellular force generation, we modulated the gel stiffness (from 4–60 kPa) by varying the ratio of acrylamide to the crosslinker, bis, but maintaining the same FN concentration and measured the forces exerted by wild-type TAF and EGFP-palladin cells. Wild-type cells exerted higher forces on gels of increasing stiffness, saturating at very high stiffness, indicating mechanosensitivity over this range of substrate compliance (Fig. 3D). We verified that EGFP-palladin expression did not affect the overall mechanosensitivity of TAF cells. The traction forces exerted by EGFP-palladin cells were similar to those of WT cells and showed a similar increase with gel stiffness (Fig. 3D). Cell shape and actin organization were also modulated by the gel stiffness. On the softest gels (Fig. 3E shows an example of a cell on a 2 kPa gel), the cell shape was rounded with a smaller area, fewer actin stress fibers and more disorganized actin structures. On stiffer gels, cells spread to a greater extent and formed robust stress fibers (Fig. 3F shows an example of a cell on a 25 kPa gel). This qualitatively underscores the fact that actin organization is correlated with cellular force generation.

### Palladin modulates cellular traction forces and mechanosensitivity

**Figure 4:**
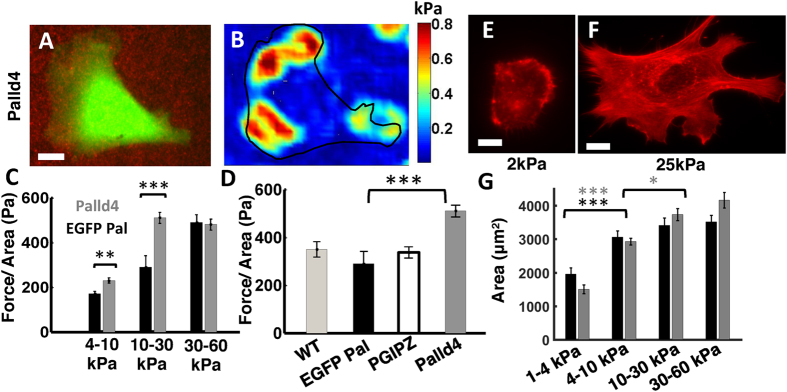
Palladin modulates cellular traction forces and mechanosensitivity. **(A)** Snapshot of a Palld4 cell on elastic gel embedded with beads. Scale bar: 10 μm. **(B)** Snapshot of traction stress map with color values corresponding to different stress values. Cell contour is superimposed in black. **(C)** Total traction force per unit area of Palld4 cells shown in the same plot with EGFP-palladin cells for comparison. (*p* < 0.01 for 4–10 kPa, *p* < 0.001 for 10–30 kPa, Wilcoxon ranksum test). Each bar represents an average of data obtained from *N* = 30–40 cells from ~6 independent experiments per condition. **(D)** Bar graph for comparison between traction stresses exerted by different cell types on intermediate stiffness (10–30 kPa) gels, showing that shRNA control cells (PGIPZ) exert similar stresses as WT and EGFP palladin cells. (*p* < 0.001, Wilcoxon ranksum test for comparison between EGFP-palladin and Palld4 cells). Each bar represents an average of data obtained from *N* > 20 cells from ~5 independent experiments. Rhodamine-phalloidin staining of a Palld4 cell on **(E)** a soft (2 kPa) gel and **(F)** a stiff (25 kPa) gel. Scale bar: 10 μm **(G)** Bar graphs comparing the spread areas of EGFP-palladin (black) and Palld4 (gray) cells as a function of gel stiffness. (*p* < 0.001, Wilcoxon ranksum test, *N* > 20 cells in each bar for comparison between 1–4 kPa and 4–10 kPa, 10–30 kPa, 30–60 kPa gels; only first comparison shown in graph; p < 0.05, Wilcoxon ranksum test, N>20 cells for comparison between 10–30 kPa and 30–60 kPa for Palld4 cells).

To examine how absence of palladin affects cell-generated forces, we imaged Palld4 cells expressing cytoplasmic GFP as a marker of stable transfection^35^ (Fig. 4A). The traction stress map (Fig. 4B), shows that Palld4 cells exert higher stresses than EGFP-palladin cells for a given stiffness range, as indicated by higher stress values. We further found that Palld4 cells also exhibited sensitivity to substrate stiffness as they generated larger forces on stiffer surfaces. However, the nature of this mechanosensitivity was different from EGFP-palladin and WT cells. The traction stresses exerted by Palld4 cells increased from soft to intermediate stiffness gels, but showed no further increase on the stiffest gels. The average stress (force per unit area) exerted by Palld4 cells was significantly higher (almost double) than those exerted by EGFP-palladin cells in the intermediate stiffness range 10–30 kPa (Fig. 4C). These data indicate that Palld4 cells are more sensitive to substrate stiffness in a lower range of stiffness as they exert larger forces and the exerted force plateaus at a lower stiffness than for control cells.

We confirmed that the effect of palladin knockdown was specific as control shRNA cells (PGIPZ) exerted similar forces as WT and EGFP-Palladin cells with no significant difference in the forces between these cells (Fig. 4D). As with WT cells, we found that on very soft gels cells spread less and lacked actin stress fibers (Fig. 4E shows an example Palld4 cell on 2 kPa gel), while on stiffer gels (25 kPa) cells had a larger spread area and numerous stress fibers (Fig. 4F shows an example Palld4 cell on a 25 kPa gel). The spread area of Palld4 cells (grey bars) was significantly smaller for the softest gels (1–4 kPa) compared to all stiffer gels (4–10 kPa, 10–30 kPa, 30–60 kPa), similar to the results with EGFP-palladin cells (black bars) (Fig. 4G). However, for the different cell types, we measured similar areas in all the ranges of substrate stiffness (ranging from 4 kPa–60 kPa) where tractions were measured, and cells formed stress fibers in this range of stiffness. These results indicate that the difference in traction stress between EGFP-palladin and Palld4 cells on soft and intermediate stiffness gels is not merely due to a difference in spread area but rather due to a change in intrinsic force generation capacity of the cell.

### Effect of palladin on myosin-based force generation

**Figure 5:**
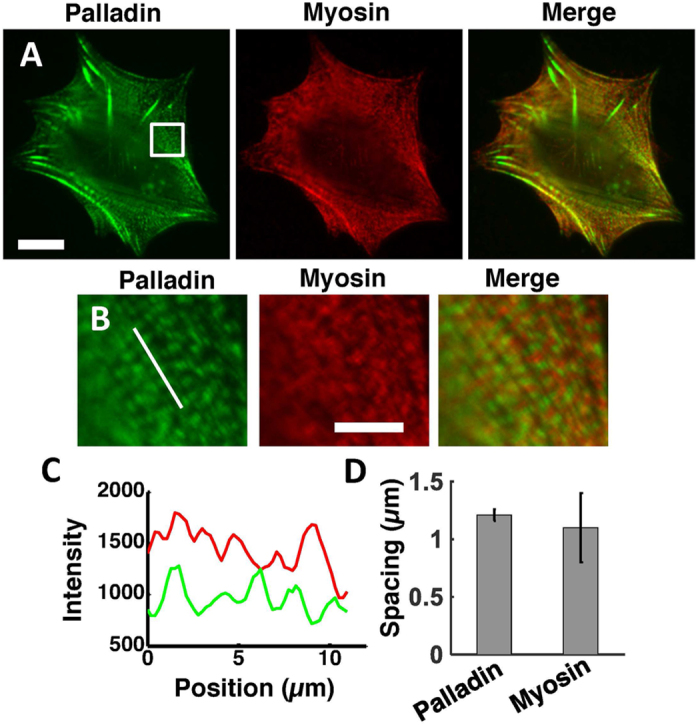
Palladin and myosin localize in alternate bands across actin stress fibers. **(A)** Widefield fluorescence image of EGFP-palladin cell labeled with mCherry-myosin-IIA showing localization of palladin (green) and myosin (red) in a spread cell. Scale bar: 15 μm. **(B)** Magnified image of the square highlighted in (A) showing alternating bands of palladin (green) and myosin (red) along actin stress fibers. Scale bar: 5 μm. **(C)** Intensity profile of the line highlighted in (**B**) showing alternating intensity peaks of palladin (green, lower profile) and myosin (red, upper profile) fluorescence. **(D)** Bar graph comparing the band spacing for palladin and myosin.

Actomyosin networks are known to be important for cellular traction force generation [51]. Myosin localizes to stress fibers in a striated pattern resembling sarcomeres in striated muscle, which suggests a role for myosin contractility in force transmission across the cell. To examine the relative localization of myosin and palladin, we transfected EGFP-palladin cells with mCherry-myosin-IIA and obtained fluorescence images of EGFP-palladin (green) and mCherry-myosin-IIA (red) (Fig. 5A). Palladin localized in a striated pattern on stress fibers (Fig. 5B) similar to myosin. Intensity profiles along stress fibers showed that myosin and palladin localized to alternate bands (Fig. 5C). For selected regions of stress fibers, we calculated the correlation coefficient between the intensities of the two line profiles. While it varied throughout the cell, in regions with dense stress fibers the correlation coefficient was significantly negative (C = −0.39, p = 0.02), indicating that palladin and myosin puncta are largely anti-correlated. For comparison, in areas away from stress fibers, C = 0. The band spacing, measured as the distance between peaks in the intensity profiles, was 1.1 ± 0.3 μm for myosin, similar to the spacing 1.21 ± 0.05 μm, of palladin bands (Fig. 5D). Cells on gels of intermediate stiffness exhibited smaller band spacing in EGFP-Palladin cells compared to Palld4 cells (Supplementary Fig. S3.) These observations are consistent with previous studies showing the localization of myosin and α-actinin in alternate bands across stress fibers^47^, since α-actinin and palladin co-localize. This periodic appearance and close proximity of myosin and palladin as well as changes in band spacing in Palld4 cells led us to hypothesize that palladin expression may have an effect on myosin’s ability to generate contractile stresses on actin networks.

**Figure 6:**
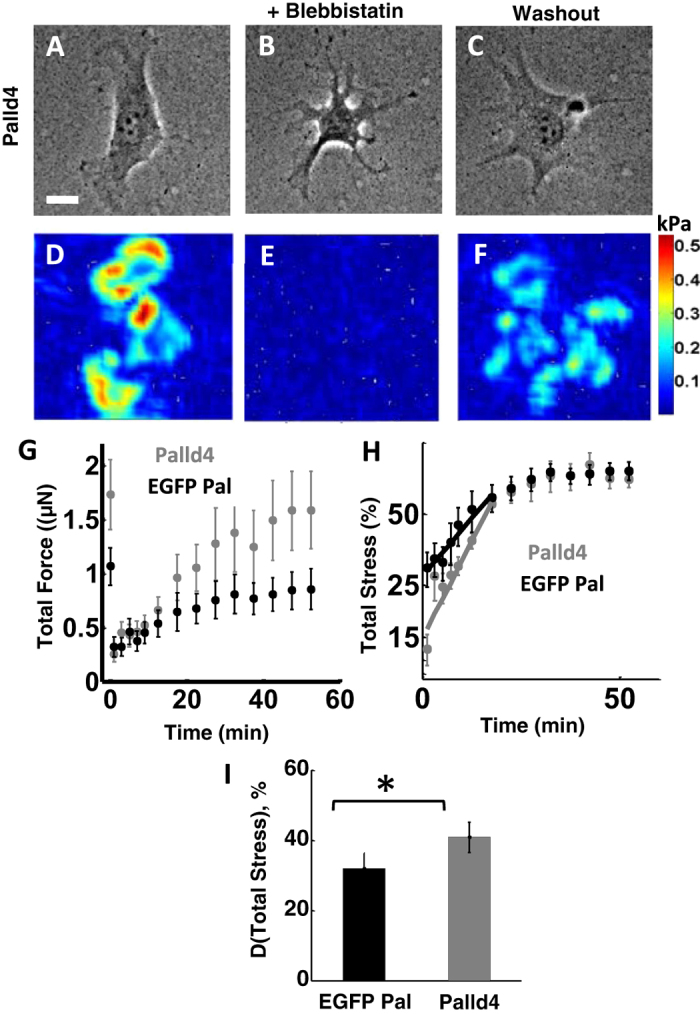
Palladin knockdown cells show more efficient recovery from Blebbistatin treatment. **(A)** DIC image of a palladin KD (Palld4) cell on a gel of intermediate stiffness (10–30 kPa range). Scale bar: 10 μm. **(B)** DIC image of the same cell as in A, 30 minutes after incubation in 15 μM blebbistatin. **(C)** DIC image of the cell 1 hour after washout from blebbistatin, showing recovery of cell morphology. **(D)** Traction force map of the cell in A showing robust generation of traction forces. **(E)** Traction force map of the cell in B, showing disappearance of traction forces upon blebbistatin addition. **(F)** Traction force map of the cell in C, showing recovery of traction forces 1 hour after Blebbistatin washout. **(G)** Total force as a function of time after removal of blebbistatin for GFP-palladin (black) and Palld4 (grey) cells. Each data point is an average of forces from N = 10 cells from independent experiments for each condition. The first data point represents the initial (pre-blebbistatin) force. The graphs show the increase in cellular traction forces as the cell recovers from blebbistatin washout, subsequent to 30 min incubation in Blebbistatin. **(H)** The percentage force (with respect to original forces) during recovery from blebbistatin after washout plotted as a function of time for EGFP-palladin cells (black) and Palld4 cells (grey). **(I)** The percentage increase of stress after washout of blebbistatin quantified as the difference between force recovered 1 hour after washout, *F*_*recov*_, and the force after incubation in blebbistatin for 30 minutes, *F*_*blebb*_ (with respect to the initial force prior to blebbistatin addition). The data represents an average for 20–30 cells of each type. (*p* < 0.05, Wilcoxon ranksum test).

To examine the role of palladin in the generation of contractile stresses, we dynamically perturbed myosin contractility using blebbistatin, a small molecule inhibitor of myosin II. Blebbistatin treatment results in loss of stress fibers, focal adhesions and traction force generation in a reversible manner^47^. We used blebbistatin to inhibit myosin II activity and quantified the recovery of force upon removal of blebbistatin (see Methods for details). We used gels of the intermediate stiffness range (10–30 kPa) as this stiffness yielded the greatest difference in forces between Palld4 and EGFP-palladin cells. Upon treatment with blebbistatin, stress fibers disassembled and cells drastically changed their shape, shrinking and leaving behind retraction fibers as shown in a representative Palld4 cell (Fig. 6A,B). After washout of blebbistatin, most cells recovered their shape (Fig. 6C) and partially recovered their stress fibers. Traction stresses dropped almost entirely upon incubation with blebbistatin for 30 min (Fig. 6D before and 6E after inhibition) and then largely recovered 60 min after washout (Fig. 6F). As seen from the plots of force recovery versus time, a large fraction of the force recovered during the first 20 minutes after blebbistatin washout (Fig. 6G).

Subsequently, the forces recovered by palladin KD cells continued to increase, while those of EGFP-palladin cells plateaued. The absolute forces recovered by KD cells were larger than those by EGFP-palladin cells, as expected since KD cells exerted higher forces before inhibition. To quantify the relative values of forces recovered, we calculated the ratio of recovered force to the initial force (before blebbistatin treatment) for each cell to obtain the percentage recovery with respect to the initial force. While the reduced value of force after blebbistatin treatment was higher for KD cells than for EGFP-palladin cells, the percentage drop in force for KD cells was greater (~15% of the original) than for EGFP-palladin cells (~30% of the original). Palladin KD cells showed a faster force recovery at early times (first 20 min during which most of the force builds up) as compared to EGFP-palladin cells indicated by a larger slope (Fig. 6H). We quantified the increase in force after blebbistatin washout as the difference (D) between the force 60 min post-recovery and at the time of blebbistatin removal as a percentage of the original force. The extent of force recovery was higher for Palld4 cells than EGFP-palladin cells, again indicating more efficient force recovery in Palld4 cells (Fig. 6I). These results indicate that lower expression of palladin facilitates recovery from the effects of myosin inhibition and that lower palladin levels are correlated with higher force generation.

### Palladin modulates speed of retrograde flow

**Figure 7:**
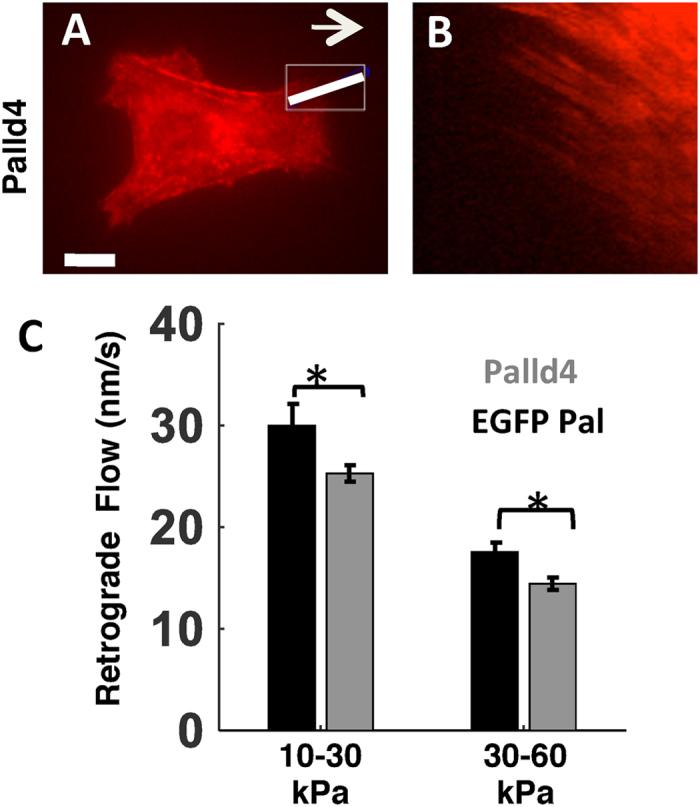
Palladin involvement in retrograde flow. **(A)** Fluorescence image of a Palld4 cell expressing mCherry-myosin. Scale bar 10 μm. **(B)** Kymograph generated along the line drawn in A showing retrograde flow of myosin which appears as red linear streaks. **(C)** Comparison of the retrograde flow speed for EGFP-palladin and Palld4 cells for different gel stiffness. Each bar represents the average of ~100–200 tracked lines along kymographs similar to the one in B (p < 0.01, t-test).

The coordination of actin dynamics and myosin II activity in lamellar and lamellipodial networks results in a continuous retrograde flow of actin, myosin, and other crosslinkers from the cell periphery towards the center^48^. Retrograde flow speed is determined by actin assembly and disassembly kinetics and myosin motor activity, and may be sensitively related to the forces generated^49^. Previous studies have shown that traction stresses are correlated with retrograde flow speeds in lamellipodia^50^. We examined the effect of palladin expression on retrograde flow in TAFs by quantifying the flow speed for EGFP-palladin and Palld4 cells spread on gels of different stiffness. Cells were transfected with mCherry-myosin, plated on fibronectin-coated gels, and imaged for 30 min to observe the centripetal flow of myosin and palladin (Fig. 7A). Kymographs along radial lines parallel to the flow show fluorescent streaks corresponding to the movement of myosin structures (Fig. 7B). The slopes of these streaks yield the flow speed (Fig. 7C). The retrograde flow speed was smaller for Palld4 cells than for EGFP-palladin cells for both stiffness ranges examined. The retrograde flow rate also varied with substrate stiffness. For both cell types, flow rates were higher on intermediate stiffness gels, while the forces exerted on these gels were smaller. This suggests an inverse dependence between traction force and retrograde flow. This is in agreement with the previously reported biphasic dependence^50^ of retrograde flow on traction forces since our observed speeds (15–30 nm/s) are in the higher flow phase of the biphasic dependence curve.

## Discussion

Here we examined the role of the actin crosslinking protein, palladin, in cell mechanics. Our results show that palladin plays a critical role in cellular force generation and mechanosensing. Palladin is essential for the efficient formation of radial stress fibers consistent with prior results in osteosarcoma cells^20^. We have previously shown that palladin knockdown cells have decreased Rac activity^51^. Conversely, increased Rac activity was shown to result in an increased number of radial stress fibers^52^, which correlates with our result of increased RSF in EGFP-palladin cells (cells with higher Rac activity) as compared to cells lacking palladin. We also found that reduced expression of palladin affects focal adhesion maturation leading to smaller adhesions which turnover more rapidly. Importantly, we found that palladin knockdown increases the force generating capacity of cells, facilitates the rapid buildup of tension within the lamellar actin network but impairs the ability to sense substrate rigidity for stiff gels. We found that cells had slower retrograde flow rates on stiffer surfaces and consistently that palladin KD cells exhibit slower flows as compared to EGFP-palladin cells. These indicate that slower flows are associated with greater traction forces implying that palladin enhances myosin-mediated actin flows on soft substrates, which result in smaller traction forces. Overall, our findings indicate that the relationship between local changes in cell response e.g. actin flow, focal adhesion dynamics and actin organization can enable the cell to sense and adapt globally to material parameters of the environment such as substrate stiffness.

Our finding that palladin knockdown cells exert forces that are almost twice as large as those exerted by wild type cells is consistent with previous studies showing that α-actinin knockdown also results in higher forces^20^,^21^. We found that the loss of radial stress fibers, decreased retrograde flow and altered focal adhesion lifetimes that accompany the loss of palladin, reduces the sensitivity of cells to sense substrate stiffness, suggesting that palladin plays a role in cellular mechanosensing. To obtain deeper insight into the mechanisms involved, we dynamically inhibited myosin activity and examined the subsequent recovery of forces after removal of inhibitor in cells with normal and reduced levels of palladin. Palladin knockdown cells showed a greater rate of force recovery, indicating that lower expression of palladin facilitates more efficient force generation by myosin. The modulation of traction stresses and kinetics of force recovery suggest that palladin expression may modulate the behavior of the actomyosin network in cells. Experiments have shown that knockdown of palladin is correlated with higher activation levels of Rho in cells (Goicoechea, unpublished). Since activated Rho is a positive regulator of myosin activity in cells, this may provide a potential link between palladin expression and myosin-based force generation.

Based on previous studies and our observations, we propose the following qualitative model of stress fiber contraction and force generation. Typically, three types of stress fibers are observed in adherent cells including TAFs – ventral stress fibers, which span the entire cell and lie along the base of the cell, radial stress fibers which are attached at one end to focal adhesions, and transverse fibers, with a sarcomeric structure, which are not attached to focal adhesions^53^,^54^. Ventral stress fibers are attached to focal adhesions at each end and show a graded polarity of actin filaments, and hence are most likely associated with contractile force generation in cells^53^,^54^,^55^,^56^. For fibers with graded polarity to contract, they should be able to displace α-actinin and palladin relative to each other and along the filaments^57^. In a proposed model^53^,^54^, actin filaments are able to contract because of the rapid association/dissociation rate of α-actinin.

**Figure 8:**
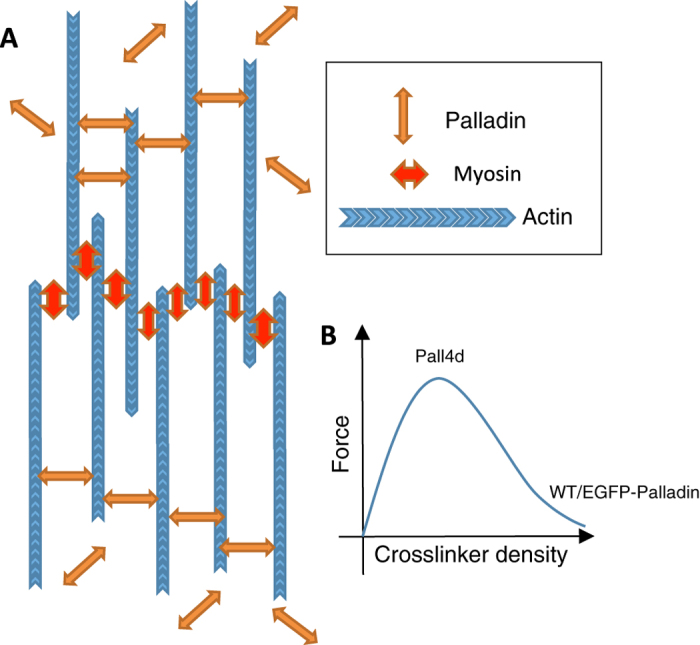
Model showing the putative role of palladin in stress fiber assembly and force generation. **(A)** Schematic representation of the proposed model. For actin filaments to move past each other, actin crosslinkers, here indicated as palladin, need to detach from the filaments. **(B)** Prediction for the dependence of force on cross-linker density.

In our representation of the proposed model, (Fig. 8A), the displacement of actin crosslinkers (e.g. palladin), which is modulated by their association/dissociation kinetics, is required for actin filaments to contract. High concentrations of crosslinkers (such as those in WT or EGFP-Palladin cells) would restrict filaments from sliding past each other and stiffen the stress fiber, resulting in decreased contraction and force generation. Conversely, lower expression levels of cross-linkers (i.e. palladin knockdown cells) would lead to greater force generation as observed. Such a mechanism may enable cells to regulate force generation by adjusting the overall concentrations of actin crosslinkers. However, a very low concentration of crosslinkers is not optimal^58^, as in this situation filaments can slide past each other without significant exertion of forces. This model, therefore, predicts a biphasic dependence of contractility on crosslinker density (Fig. 8B). The right branch of the curve represents the crosslinker levels in the WT or the EGFP-Palladin cells whereas the middle branch would represent the crosslinker levels in the Palld4 cells.

Active matter theory predicts the general sigmoidal form of the force v/s stiffness relationship^59^. In this model, the relevant parameters are the force capacity of the cell, which likely corresponds to the expression level and activity of myosin motors and the internal stiffness of the cellular cytoskeleton, relative to the substrate stiffness. In this model, lowering the level of cross-linkers would correspond to a steeper transition to the saturating force. Thus, the difference between WT and KD cells would be most apparent at intermediate stiffness.

Actin crosslinkers and myosin may be present in an optimum concentration for proper force generation and mechanical response. Exertion of forces that are too large may hinder cells’ ability to discriminate between different mechanical properties of substrates, as forces need to be tuned closely to match the cell surroundings. In summary, the differences in cellular contractility arising from palladin expression levels suggest that palladin is involved in many aspects of cell mechanics. Its interaction with myosin motors may serve as a foundation for traction force regulation. Understanding the molecular mechanisms underlying palladin’s involvement in cellular forces and mechanical sensing, in particular the role of its interactions with other actin crosslinkers such as alpha-actinin, will be a topic of our future studies.

## Additional Information

**How to cite this article**: Azatov, M. *et al*. The actin crosslinking protein palladin modulates force generation and mechanosensitivity of tumor associated fibroblasts. *Sci. Rep.* **6**, 28805; doi: 10.1038/srep28805 (2016).

## Supplementary Material

Supplementary Information
