# Supplementary material for: The actin crosslinking protein palladin modulates force generation and mechanosensitivity of tumor associated fibroblasts
Source: Sci Rep. 2016 Jun 29;6:28805. doi: 10.1038/srep28805 (PMC4926206; doi:10.1038/srep28805)
Supplement: Supplementary Information [file srep28805-s1.doc]

**The actin crosslinking protein palladin modulates force generation and mechanosensitivity of tumor associated fibroblasts**

Mikheil Azatov^1^, Silvia Goiccochea^2^, Carol Otey^3^ and Arpita Upadhyaya^1,4*^

^1^ Department of Physics, University of Maryland, College Park MD 20742, USA, ^2^Department of Biological Sciences, University of Toledo, Toledo, Ohio, 43606, USA, ^3^Department of Cell Biology and Physiology and the Lineberger Comprehensive Cancer Center, University of North Carolina at Chapel Hill, Chapel Hill, NC 27599, USA, ^4^Institute for Physical Science and Technology, University of Maryland, College Park, MD 20742, USA


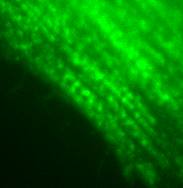

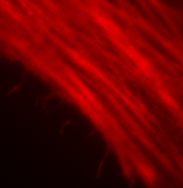

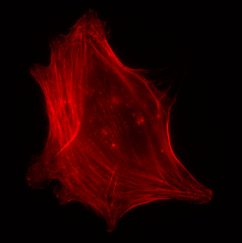

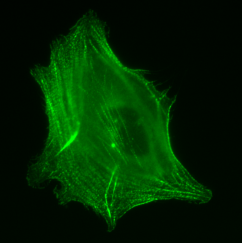

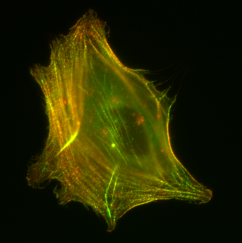


**A**

**μm**

**C**

**B**

**Supplementary Figure S1: Palladin organization in cells. (A)** Snapshots of a fully spread cell showing that EGFP-Palladin (left panel) and actin (middle panel, as visualized by Rhodamine-phalloidine staining) are colocalized (right panel) in stress fibers. This confirms what has been shown before in the literature, and indicates that the expressed EGFP-Palladin behaves in an expected manner. Scale bar: 20 µm **(B)** Zoomed in image of an actin stress fiber in an EGFP-Palladin cell showing the localization of palladin (green, left panel) and actin (red, right panel) across a stress fiber. Scale bar: 10 µm **(C)** The intensity profile across the line shown in (B, right panel) proving the periodicity of palladin bands (green) and the smoother intensity profile of actin (red).


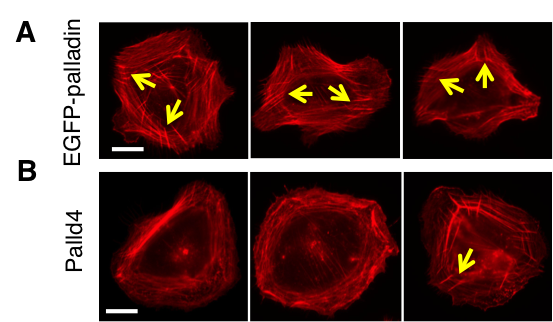


**Supplementary Figure S2: Radial Stress Fibers in TAF cells. (A)** Three examples snapshots of EGFP-palladin cells labeled with Rhodamine-phalloidin for actin showing strong radial fibers (RSF). Some examples of RSF are indicated by the arrows. Scale bar: 15 µm. **(B)** Snapshots of three example palladin KD (Palld4) cells labeled with Rhodamine-phalloidin showing fewer radial stress fibers in the cell. Scale bar: 15 µm.


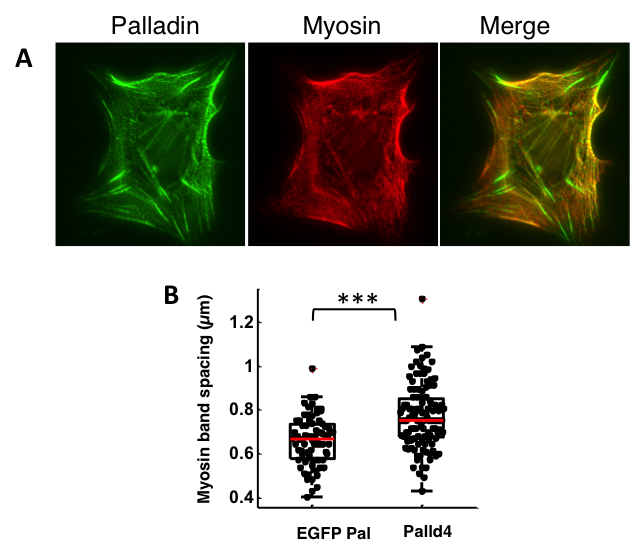


**Supplementary Figure S3: Palladin and myosin localization. (A)** Representative images of cells expressing EGFP-Palladin and mCherry-myosin.  **(B)** Beeswarm graphs showing comparison of the myosin band spacing in EGFP-palladin and palladin KD cells on elastic substrates (p<0.00001, Wilcoxon ranksum test, substrate stiffness ~ 15 kPa).
